# Irradiation-Induced Synthesis of Ag/ZnO Nanostructures as Surface-Enhanced Raman Scattering Sensors for Sensitive Detection of the Pesticide Acetamiprid

**DOI:** 10.3390/s22176406

**Published:** 2022-08-25

**Authors:** Po-Tuan Chen, Yu-Chun Lu, Sripansuang Tangsuwanjinda, Ren-Jei Chung, Rajalakshmi Sakthivel, Hsin-Ming Cheng

**Affiliations:** 1 Department of Vehicle Engineering, National Taipei University of Technology, Taipei 10608, Taiwan; 2 Department of Chemical Engineering and Biotechnology, National Taipei University of Technology, Taipei 10608, Taiwan; 3 Department of Electronic Engineering, Ming-Chi University of Technology, New Taipei City 243, Taiwan; 4 Organic Electronics Research Center, Ming-Chi University of Technology, New Taipei City 243, Taiwan

**Keywords:** surface-enhanced Raman scattering, Ag nanoparticles, acetamiprid detection, irradiation synthesis

## Abstract

Detecting pesticides using techniques that involve simple fabrication methods and conducting the detection at very low levels are challenging. Herein, we report the detection of acetamiprid at the quadrillionth level using surface-enhanced Raman scattering (SERS). The SERS chip comprises Ag nanoparticles deposited on a tetrapod structure of ZnO coated onto indium tin oxide glass (denoted as Ag@ZnO-ITO). Controlled Ag decoration of ZnO occurs via irradiation-induced synthesis. The morphology of the surface plays a significant role in achieving an enhanced SERS performance for acetamiprid detection. 4,4′-Dipyridyl (DPY) is used to investigate synthesis conditions for the chip, leading to an optimal irradiation time of 60 min. Furthermore, the enhancement factor for acetamiprid on Ag@ZnO-ITO is higher than 10^7^. These results demonstrate that SERS sensors have the potential for practical use in acetamiprid detection.

## 1. Introduction

Since the last century, pesticides have been used extensively as chemical reagents to manufacture agricultural products. Pesticide residues formed by their excessive use and their chemical and physical properties have caused harmful environmental and human health issues [1,2,3]. Acetamiprid is among the most used pesticides, and its residues in vegetables can have slight, acute, or adverse effects on humans [4,5]. Residues interfere with the functioning of the human central nervous system, contributing to numerous diseases such as Alzheimer’s disease, depression, schizophrenia, and Parkinson’s disease [6]. Therefore, several strategies for the rapid detection of acetamiprid have been explored [7,8,9]. However, most methods have limitations, such as cumbersome sample preparation steps, low detection sensitivity, and requirement of large amounts of organic solvents and costly equipment. Therefore, the development of quicker, highly sensitive, and more reliable techniques than existing methods for acetamiprid detection is imperative to improve food safety and protect the environment [10,11].

Noble metal nanomaterials (e.g., Au and Ag) have been widely used in surface-enhanced Raman scattering sensors (SERS). The principle of electromagnetic field improvement together with the adsorption of molecules on the surface leads to Raman enhancement [12,13]. Generally, SERS beacon molecules encrypt unique nanoparticles (NPs) owing to their sensitivity to a single molecule and their specificity associated with molecular fingerprints that can deliver exclusive optical features for detection. Moreover, SERS can effectively analyze molecules in their native location and is unaffected by the sample background and detection mechanism. Therefore, SERS has emerged as an essential detection method for biosensor applications [14,15,16]. Metallic NPs demonstrate dependent properties, also called surface plasmon resonance (SPR) [17,18]. Nevertheless, SERS-encoded NPs characteristically display a low-intensity signal, and the irrepressible aggregation of NPs results in SERS signals that cannot be reproduced, limiting their application. Hence, a well-regulated nanostructure assembly is important for improving the reproducibility of detection using SERS [19,20].

In particular, Ag@ZnO has been studied for application in SERS because of its unique sensitivity and selectivity [21,22]. ZnO tetrapod nanostructures have been used as electrode templates because of their four-pillar structure, which has a larger surface area than that of other structures [23,24,25]. The electrophoretic deposition method for ZnO synthesis on an indium tin oxide (ITO) substrate can effectively allow for the low-cost, continuous mass production of high-purity, non-aggregated ZnO powder [26]. Various nanostructured materials have been decorated on ZnO, and these heterogeneous materials have shown the ability to rapidly transfer electrons [27]. Moreover, Ag NPs have excellent Raman response activity, and ZnO tetrapod supports show an increased enhancement effect owing to the coupling of porous ZnO and plasmon resonance of Ag NPs [28,29].

Ultrasensitive, stable, facile, and quick detection remains a challenge for acetamiprid sensors using the SERS technique. Here, the practical application of a highly sensitive SERS-based chemosensor for acetamiprid is reported. The preparation of Ag@ZnO-ITO via photoirradiation-induced synthesis [30,31] is demonstrated. Irradiation is a convenient method for the synthesis of controllable Ag nanostructures on SERS chips. An SPR-mediated collective oscillation of transfer electrons can be produced on the Ag NP surface, causing an improved electric field to be generated locally [32]. We use 4,4′-dipyridyl (DPY) to determine the parameters of the irradiation time because DPY and acetamiprid have SERS-sensitive pyridine functional groups, and the SERS effect of DPY on Ag@ZnO has been extensively investigated [33]. After determining the optimal time for irradiation-induced synthesis, we use the chips for SERS. The enhancement factor for acetamiprid is higher than 10^7^. The high sensitivity and reliability of a real sample assay are demonstrated using the as-developed rapid and simple SERS-based chip sensor, which introduces a novel method for the fabrication of SERS-based pesticide detectors.

## 2. Materials and Methods

### 2.1. Fabrication of Platinum Anode for Electrochemical Processes

In the electrochemical process, platinum (Pt) glass served as the anode. To prepare the anode, first, the conductive ITO glass (dimensions: 2.6 × 3.5 mm) was cleaned and sequentially washed with deionized water (DD H_2_O), acetone, and isopropanol (IPOH) for 10 min each in an ultrasonic bath. After cleaning, the ITO plates were dried for 5 min at an ambient temperature of approximately 26 °C. Subsequently, 0.3 mM dihydrogen hexachloroplatinate (IV) was employed as the source of Pt, which was deposited on the conductive ITO via spin coating at 800 rpm (at ambient temperature) for 60 s. Thereafter, the Pt-deposited chip was placed on a plate and maintained at 400 °C for 2.5 h to obtain a dense and smooth surface of Pt nanocrystals. Finally, the fabricated electrodes were cleaned with IPOH and DD H_2_O prior to drying them at ambient temperature.

### 2.2. Synthesis of Ag@ZnO-ITO SERS Chips

An electrophoretic coating method was used to prepare the ZnO suspension under galvanostatic conditions [26]. ZnO powder (0.35 g) was added to a 10 mL mixture of butanol/IPOH/ethanol (volume ratio 4/2/1) and then sonicated for 30 min. A Pt substrate and cleaned ITO glass were used as the anode and cathode, respectively, and the substrates were dipped vertically into the suspension. A direct current (DC) power supply of 10 V was fixed at a distance of 7 mm between the anode and cathode, resulting in a current of approximately 100 μA. The voltage was applied for 3 min, resulting in a ZnO layer of ~10–15 μm thickness. Following this, the ZnO chip was sintered at a temperature of 390 °C for 3.5 h.

The Ag@ZnO surface was prepared using a photoirradiation-induced synthesis method, which provided a more efficient and uniform Ag coverage than precipitation, dipping, or electrodecoration [29,30]. Briefly, the as-prepared ZnO chip was placed in 50 mL of a 0.1 M AgNO_3_ aqueous solution. The chip was placed vertically, and the side with ZnO was oriented to face the subsequent light exposure. A 300 mm distance between the chip and the center of the light source was maintained, and the solution was continuously stirred to ensure a homogeneous reaction. A universal handy-type ultraviolet (UV) lamp with a wavelength of 365 nm was used for irradiation. The ZnO-ITO film was subjected to UV irradiation-induced synthesis from 0 to 90 min for the deposition of Ag NPs. The Ag@ZnO-ITO films were rinsed with DD H_2_O several times and dried at room temperature in the dark. After cleaning, a hot air blower (at ~50 °C) was used for 10 min to dry the SERS chips.

### 2.3. Raman Detection

A DPY solution was prepared as a reference to examine the performance of the Ag@ZnO-ITO films fabricated at various irradiation times. Micro-Raman spectroscopy was performed using a 785 nm laser with a power of 25 mW and a 10× objective lens with a spot size of 16 μm^2^. The Raman spectroscopy was performed for approximately 10 s per scan at each concentration. All experiments were repeated five times.

The ultrasensitive detection of acetamiprid was also investigated. Acetamiprid solutions of different concentrations were prepared in 20 mL. Thereafter, 10 mL of the sample solution were uniformly drop-cast onto the SERS chip using a pipette. Raman spectra were obtained using a 532 nm laser at 25 mW power and 10× magnification.

### 2.4. Instruments

For organic matter, quantitative analysis was performed using gas chromatography-mass spectrometry (GC-MS, Shimadzu TQ8040, Shimadzu Corporation, Kyoto, Japan). The crystallinity of Ag@ZnO-ITO was examined using powder X-ray diffraction (PXRD, Bruker D8 Advance, Bruker Corporation, Billerica, MA, USA). The surface morphology of Ag@ZnO-ITO was probed using field-emission scanning microscopy (FE-SEM, JEOL FSM-7610F plus, JEOL, Ltd., Tokyo, Japan). Crystallinity calculations and Raman measurements were performed using FullProf Suite (Version 7.30, Mar 2020-ILL JRC) and micro-Raman spectroscopy (Horiba IHR 550, Horiba, Ltd., Kyoto, Japan), respectively. The ultraviolet-visible (UV-Vis) absorption spectra of the samples were recorded using a detector (Jasco RI 930, Jasco Inc., Easton, MD, USA).

## 3. Results and Discussions

### 3.1. Characterization of Ag@ZnO

**Figure 1:**
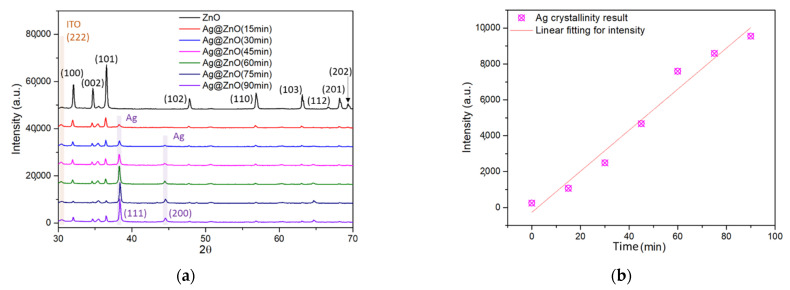
(**a**) XRD results for ZnO-ITO and Ag@ZnO-ITO at various irradiation times, and (**b**) the intensity variation of the peak at 2θ = 38.01°.

The crystallinity and chemical nature of Ag@ZnO after various irradiation times were examined using XRD analysis. The XRD patterns of ZnO and Ag@ZnO at different irradiation times are shown in Figure 1a. In general, enhancement of the Ag coverage reduces the diffraction signal of ZnO and affects the absorption and diffraction of X-rays. This signal decrement phenomenon also occurred in energy dispersive spectroscopy (EDS) analysis data. (See the comparison of Ag/Zn ratio, which is consistent with the XRD trend, in the Supporting Information Table S1). The peaks at 2θ values of 31.78°, 34.41°, 36.38°, 47.56°, 56.76°, 62.89°, 66.40°, 67.93°, and 69.03° were indexed to the (100), (002), (101), (102), (110), (103), (112), (201), and (202) planes of ZnO, respectively. The obtained peaks can be attributed to the hexagonal wurtzite structure of ZnO, which is consistent with JCPDS No. 36-1451. ITO exhibited a polycrystalline nature and crystallized in a cubic structure (JCPDS No. 71-2194) with a predominant (222) peak at a 2θ value of 30.45°. A switch in the preferential growth direction from the (400) to the (222) plane was observed when tin (Sn) was doped with indium oxide. Ag particles were decorated on the ZnO surface. Hence, the incident X-rays mostly strike the Ag nanoparticles. That is, as the deposition time of Ag increases, more Ag is decorated on the ZnO surface, which makes it the main contributor to XRD intensity. After Ag decoration, two additional peaks, at 38.01° and 44.50°, were observed in the XRD pattern of Ag@ZnO-ITO, which are likely related to the (111) and (200) planes of Ag, respectively. The diffraction peaks at 2θ values for Ag correspond to the face-centered cubic (fcc) phase of the metallic Ag (Ag0) (JCPDS No. 04-0783) [34]. No diffraction peak is observed at 2θ = 32.97°, which indicates the absence of Ag_2_O in the material. Additionally, no changes in the peak position were observed for Ag@ZnO-ITO at various irradiation times, indicating that Ag was not doped. The XRD patterns illustrate that Ag@ZnO-ITO tetrapods were successfully prepared via the irradiation synthesis method.

Standard Williamson-Hall analysis was used to assess the average crystallite size and microstrain of ZnO [35]. As stated by the fitting results considering the least-square difference, ZnO did not display any stress. After the deposition of Ag on the ZnO surface, the crystallite size of the ZnO NPs declined (from 73.6 nm to 72.8 nm). No significant decrease in the diffraction peak intensity related to distortion was observed in the lattice structure.

The intensity of the peak at 2θ = 38.01° increased with increasing irradiation time. As shown in Figure 1b, the increase in intensity with irradiation time is linear.

**Figure 2:**
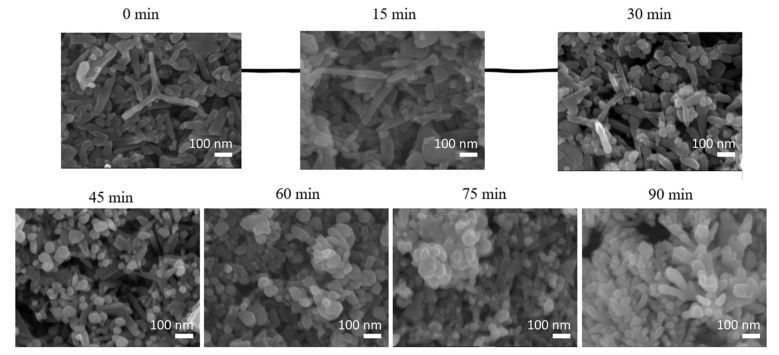
FE-SEM images of Ag@ZnO-ITO at various irradiation times.

FE-SEM was used to investigate the morphology of pure ZnO and Ag@ZnO chips at various irradiation times. As shown in Figure 2, at high magnification, the ZnO chips show stacks of ZnO tetrapod nanostructures fabricated in a porous form on an ITO substrate. The original ZnO nanostructures are polymorphic, consisting of nanopillars and branched and tetrapod-shaped ZnO powder. The axial length is approximately 100–400 nm and the radial direction is approximately 30–80 nm. At an irradiation time of 0 min, pure ZnO reveals numerous rod diameters and lengths for the individual ZnO tetrapod structures. The surface of the ZnO is very smooth.

Subsequently, a large amount of Ag adhered to the ZnO surface in the irradiation-induced synthesis of Ag NPs on the surface of the ZnO tetrapod. This suggests that agglomeration occurred and establishes that the morphology of Ag-decorated ZnO and of pure ZnO NPs is similar. This similarity is attributed to the adsorption of Ag on ZnO, rather than its substitution into ZnO. After irradiation for times ranging from 15 to 90 min, the Ag was slightly agglomerated on the ZnO surface, and it had a near-spherical shape and identical morphology. With an increase in the irradiation time, the size of the Ag NPs also increased. The smallest diameter of Ag NPs is ~8 nm at 15 min; this result suggests that individual Ag NPs aggregate to form larger Ag NPs during the decoration process via irradiation [36]. After irradiation for 90 min, the size of the Ag NPs reached 25 nm.

Highly crystalline particles exhibit well-defined images and clear lattice fringes. The XRD patterns in Figure 1 show that a wide peak occurs at twin ZnO and Ag NPs with smaller grain sizes, which renders it difficult to identify the diffraction peaks of the Ag NPs.

**Figure 3:**
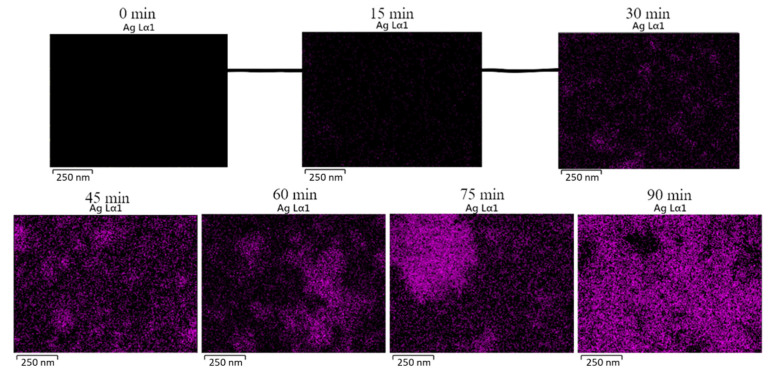
EDS mapping of Ag@ZnO-ITO at various irradiation times.

To verify the presence of Ag NPs after irradiation, the chemical composition was explored using X-ray EDS (Figure 3), which demonstrated that the Ag NPs were decorated on the ZnO-ITO after irradiation-induced synthesis. The EDS elemental map clearly shows Ag signals after an irradiation time of 15 min. The presence of Ag, signified by the purple color in the EDS mapping, demonstrates a large amount of Ag NPs on the ZnO surface. Hence, well-defined heterojunctions were obtained between the ZnO tetrapods and the Ag NPs. The Ag/Zn mole ratios at different irradiation times are presented in the Supporting Information, Figure S1 and Table S1.

### 3.2. UV-Vis Absorption Spectra

**Figure 4:**
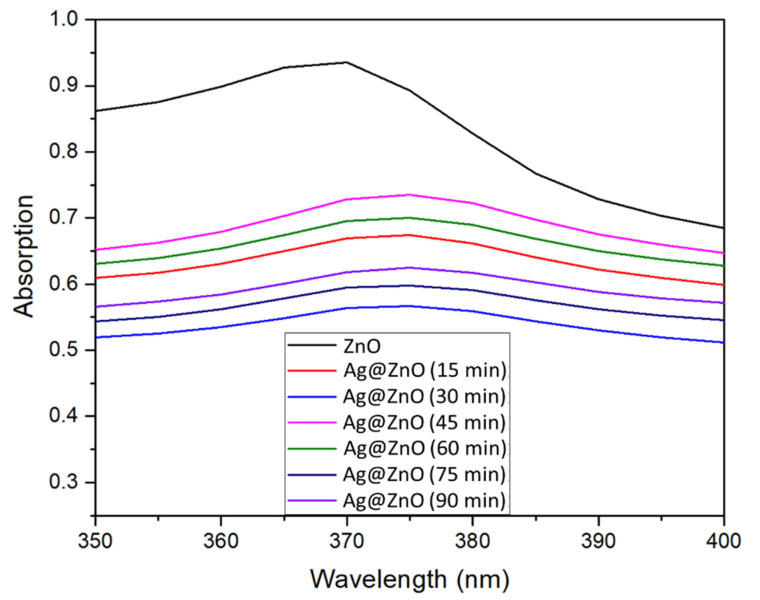
UV-Vis spectra of ZnO and Ag@ZnO at various irradiation times.

Figure 4 shows the UV-vis absorption spectra of ZnO and Ag@ZnO-ITO. ZnO exhibits an absorption peak at 370 nm, which corresponds to ZnO [37]. The absorption of Ag@ZnO-ITO is not a simple superposition of a distinct single component. Owing to the strong electronic connection between the ZnO substrate and the Ag NPs, the Ag@ZnO surface plasmon peak is significantly broadened at 375 nm [38]. There were no significant changes in the spectra with varying irradiation time. The Ag NPs on the ZnO substrate enriched the charge separation, wherein the large substrate thickness was enhanced by the refractive index surrounding the medium.

### 3.3. Irradiation-Time-Dependent SERS Activity

**Figure 5:**
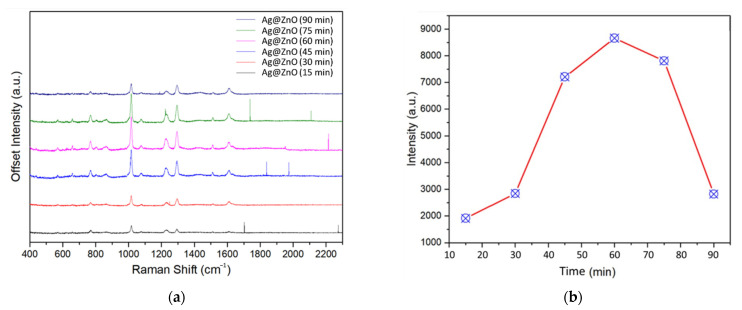
(**a**) SERS of DPY adsorbed on Ag@ZnO-ITO synthesized at various irradiation times. (**b**) Raman intensity of peak at 1298 cm^−1^ of DPY adsorbed on Ag@ZnO-ITO at various irradiation times.

Although Ag systems have been broadly examined for SERS applications, there have been few studies on the irradiation-induced synthesis of Ag NPs for the detection of acetamiprid via SERS. To inspect the dependence of SERS activity on the irradiation time, we first selected DPY as the probe molecule. The concentration of DPY used was 6.4 × 10^−7^ M. Figure 5a presents a set of DPY SERS spectra adsorbed on Ag@ZnO-ITO synthesized at various irradiation times to determine the irradiation-time-dependent SERS activity. Significantly, the strength of the signal in the Raman spectrum of DPY increases with increasing irradiation time, up to 60 min. The Raman spectral peak positions of DPY include 771, 1028, 1064, 1220, 1299, 1510, and 1612 cm^−1^ [39].

In the resonance Raman spectrum, the DPY powder showed a strong peak at 1623 cm^−1^, corresponding to the C–C stretching vibration and in-plane C–N bending vibration of the pyridyl ring. The DPY solution demonstrated a symmetry mode at 1612 cm^−1^ ascribed to the C–C stretching vibration. Similarly, the DPY powder exhibited a Raman signal at 1609 cm^−1^, which was ascribed to C–C stretching vibration. The DPY powder and solution peaks at 1513 cm^−1^ and 1510 cm^−1^ correlate to the vibration modes of C–C and C–N bonds, respectively. The DPY powder exhibited peaks at 1001 cm^−1^, 1219 cm^−1^, and 1298 cm^−1^, while the DPY solution exhibited peaks at 1064 cm^−1^, 1220 cm^−1^, and 1299 cm^−1^. These are attributed to vibrations of C–C and C–N plus the in-plane bending vibration of C–H modes, which demonstrates a strong connection between the adsorbate and the Ag NPs. Additionally, the DPY solution exhibited a peak at 771 cm^−1^, related to ring breathing vibration.

The SERS enhancement is attributed to the coupling of Ag plasmon resonance and ZnO [27]. In general, the optimal irradiation time for SERS is correlated with the size of the metallic NPs, which depends on the effects of surface scattering and radiation damping. When the particle size is smaller than the mean free path of a transmission electron, the size of the metal particles becomes dependent on the dielectric constant. Surface scattering results in a significant modification of the imaginary part of the dielectric constant, and the SERS improvement is limited through surface plasmon damping. This indicates that smaller NPs do not favor a large degree of SERS enhancement. However, dynamic depolarization and radiation damping also alter the SERS signal, which increases with increasing particle size. Figure 5b shows that the SERS intensity of the peak at 1298 cm^−1^ reached a maximum for the Ag particles synthesized with an irradiation time of 60 min. Notably, we demonstrated that irradiation-induced synthesis can effectively control the size of Ag NPs, which can be employed as an optimal SERS substrate in terms of reproducibility, sensitivity, and stability.

### 3.4. SERS Activity of DPY

**Figure 6:**
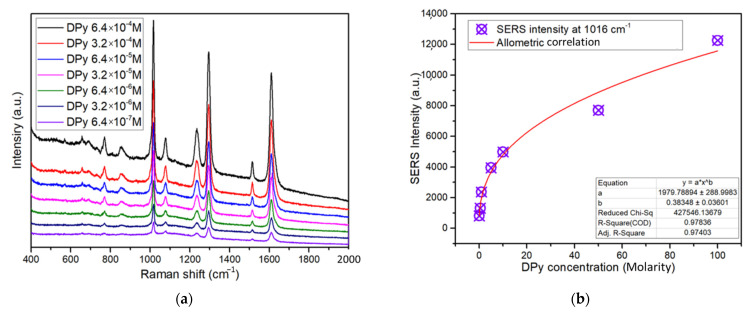
(**a**) SERS detection at various concentrations of DPY with Ag@ZnO-ITO synthesized at an irradiation time of 60 min. (**b**) *EF* in various concentrations of DPY solution.

Figure 6a shows the DPY SERS spectra for various concentrations of Ag@ZnO-ITO. The intensity of the Raman signal for DPY increases with the DPY concentration. Even when the concentration of DPY was 6.4 × 10^−7^ M, characteristic Raman spectral peaks were clearly observed.

We selected the most prominent peak for DPY and Ag@ZnO-ITO as the quantitative analysis standard after 60 min of irradiation (1299 cm^−1^). The enhancement factor (*EF*) for various DPY concentrations determined from the SERS spectra was evaluated using the following equation [40]:

(1) $$EF=\frac{N_{\left(glass\right)}\times I_{\left(Ag\right)}}{N_{\left(Ag\right)}\times I_{\left(glass\right)}},$$

where *N*(*glass*) and *N*(*Ag*) represent the number of DPY molecules on the glass substrate and Ag, respectively. *I*(*glass*) and *I*(*Ag*) denote the Raman intensities of the peaks at 1299 cm^−1^. This peak position represents strong in-plane deformation vibration. The plot of the calculated result is shown in Figure 6b, in which the *EF* of DPY is correlated with the concentration of DPY. During the addition of the DPY solution, the Ag particle distance decreased after absorption of the DPY molecules and was dependent on the DPY concentration [41]. The *EF* increased from 651 to 2.95 × 10^7^ M as the DPY concentration diminished from 6.4 to × 10^−4^ M to 6.4 × 10^−7^ M. In addition, the *EF* of DPY in logarithmic scale is presented in Supporting Information Figure S2.

### 3.5. SERS Activity of Acetamiprid

**Figure 7:**
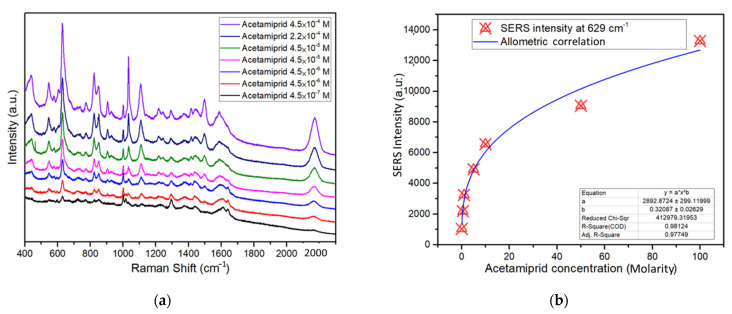
(**a**) SERS detection of various concentrations of acetamiprid with Ag@ZnO-ITO synthesized with an irradiation time of 60 min. (**b**) *EF* for various concentrations of acetamiprid.

We examined the ability of SERS to detect acetamiprid using Ag@ZnO-ITO, synthesized with an irradiation time of 60 min. As shown in Figure 7a, there was a gentle increase in the Raman signal intensity of acetamiprid with increasing acetamiprid concentration from 4.5 × 10^−7^ M to 4.5 × 10^−4^ M. When the acetamiprid concentration was 4.5 × 10^−4^ M, the feature peak of acetamiprid was observed; however, no characteristic peak of acetamiprid was detected at this concentration. Therefore, the performance of Ag@ZnO-ITO based on SERS for the detection of acetamiprid was effective.

Characteristic peaks of acetamiprid at Raman shifts of 629 cm^−1^, 854 cm^−1^, 827 cm^−1^, 1043 cm^−1^, 1109 cm^−1^, 1494 cm^−1^, and 1598 cm^−1^ were clearly observed based on SERS detection. Among the normal modes [42,43,44], the peak at 629 cm^−1^ is due to the ring structure. The peak at a Raman shift of 854 cm^−1^ is related to the C–N–C stretching. The peak at 827 cm^−1^ is derived from the ring vibration of para-disubstituted benzene, and the peak at 1043 cm^−1^ is related to the ring vibration of the ortho-disubstituted benzene. The band at 1109 cm^−1^ is related to CH in-plane deformation and C–C–C in-plane deformation vibrations, the ring breathing vibration mode, or the N–C stretching mode in the ring. The bands at 1494 cm^−1^ and 1588 cm^−1^ are ascribed to ring breathing vibrations.

We selected the most prominent peak at 629 cm^−1^ for acetamiprid and Ag@ZnO-ITO after 60 min of irradiation synthesis as the quantitative analysis standard. *EF* values for various acetamiprid concentrations were also evaluated. The calculated results are shown in Figure 7b, in which the *EF* of acetamiprid is correlated with its concentration. In addition, the *EF* of acetamiprid in the logarithmic scale is presented in Supporting Information Figure S2. Upon adding the acetamiprid solution, the Ag particle distance decreases owing to the absorption of acetamiprid molecules and the resonance spots for the acetamiprid SERS spectra. The *EF* value increased from 1000 to 2.95 × 10^7^ M as the concentration of DPY diminished from 4.5 × 10^−4^ M to 4.5 × 10^−7^ M. The limit of detection of acetamiprid using the SERS technique was determined to be 4.5 × 10^−7^ M, which is lower by a factor of seven than the optimum levels specified by the U.S. EPA in drinking water and is comparable to the examination capability reported in previous studies [45,46,47].

## 4. Conclusions

We fabricated a nanostructured composite consisting of ZnO tetrapods decorated with Ag NPs on ITO glass via irradiation-induced synthesis. Ultrasensitivity for acetamiprid detection resulting from the extremely SERS-active NPs was demonstrated down to the quadrillionth level. The irradiation-induced fabrication of a SERS chip allowed the successful decoration of the ZnO surface with Ag to obtain Ag NPs of regulated sizes which can be employed as an optimum SERS substrate in terms of reproducibility, sensitivity, and stability. The SERS intensity reached a maximum for Ag NPs synthesized with an irradiation time of 60 min. Highly efficient charge transport by controllable plasmonic metal NPs on semiconductors can advance SERS applications for detecting pesticides in the solution. These SERS chips have many advantages including specificity, recyclability, long-term stability, portability, facile operation, and cost-effectiveness. This SERS technique could be extended to establish novel avenues for establishing and advancing chemical sensing using SERS-based techniques under various conditions.

## Data Availability

Not applicable.

## Supplementary Materials

The following supporting information can be downloaded at: https://www.mdpi.com/article/10.3390/s22176406/s1. Table S1. Semi-quantitation of element from EDS spectrum of Ag@ZnO-ITO with various irradiation times. Figure S1. EDS spectrum of Ag@ZnO-ITO with various irradiation times. Figure S2. *EF* in the logarithmic scale of various concentrations of (a) DPY and (b) acetamiprid solution.
