# Supplementary material for: Irradiation-Induced Synthesis of Ag/ZnO Nanostructures as Surface-Enhanced Raman Scattering Sensors for Sensitive Detection of the Pesticide Acetamiprid
Source: Sensors (Basel). 2022 Aug 25;22(17):6406. doi: 10.3390/s22176406 (PMC9459916; doi:10.3390/s22176406)
Supplement: Supplementary file 1 [file sensors-22-06406-s001.zip › sensors-1803836-supplementary.pdf]

sensors

MDPI

Supporting Information

Irradiation-Induced Synthesis of Ag/ZnO Nanostructures as Surface-Enhanced Raman Scattering Sensors for Sensitive Detection of the Pesticide Acetamiprid

Po-Tuan Chen [1], Yu-Chun Lu [2], Sripansuang Tangsuwanjinda [3,4], Ren-Jei Chung [2], Rajalakshmi Sakthivel [2] and Hsin-Ming Cheng [3,4,*]

[1] Department of Vehicle Engineering, National Taipei University of Technology, Taipei 10608, Taiwan
[2] Department of Chemical Engineering and Biotechnology, National Taipei University of Technology, Taipei 10608, Taiwan
[3] Department of Electronic Engineering, Ming-Chi University of Technology, New Taipei City 243
[4] Organic Electronics Research Center, Ming-Chi University of Technology, New Taipei City 243, Taiwan
[*] Correspondence: smcheng@mail.mcut.edu.tw; Tel.: +886-29089899 (ext. 4867)

Table S1. Semi-quantitation of element from EDS spectrum of Ag@ZnO-ITO with various irradiation times.

Times	0 min	15 min	30 min	45 min	60 min	75 min	90 min
Zn (%)	82.5	77.6	73.3	69.4	62.0	53.3	47.2
O (%)	17.5	11.5	14.7	15.4	13.5	13.4	12.0
Ag (%)	0	10.9	12.0	15.2	24.5	33.3	40.8
Ratio of Ag/Zn	0	0.14	0.16	0.22	0.40	0.62	0.86

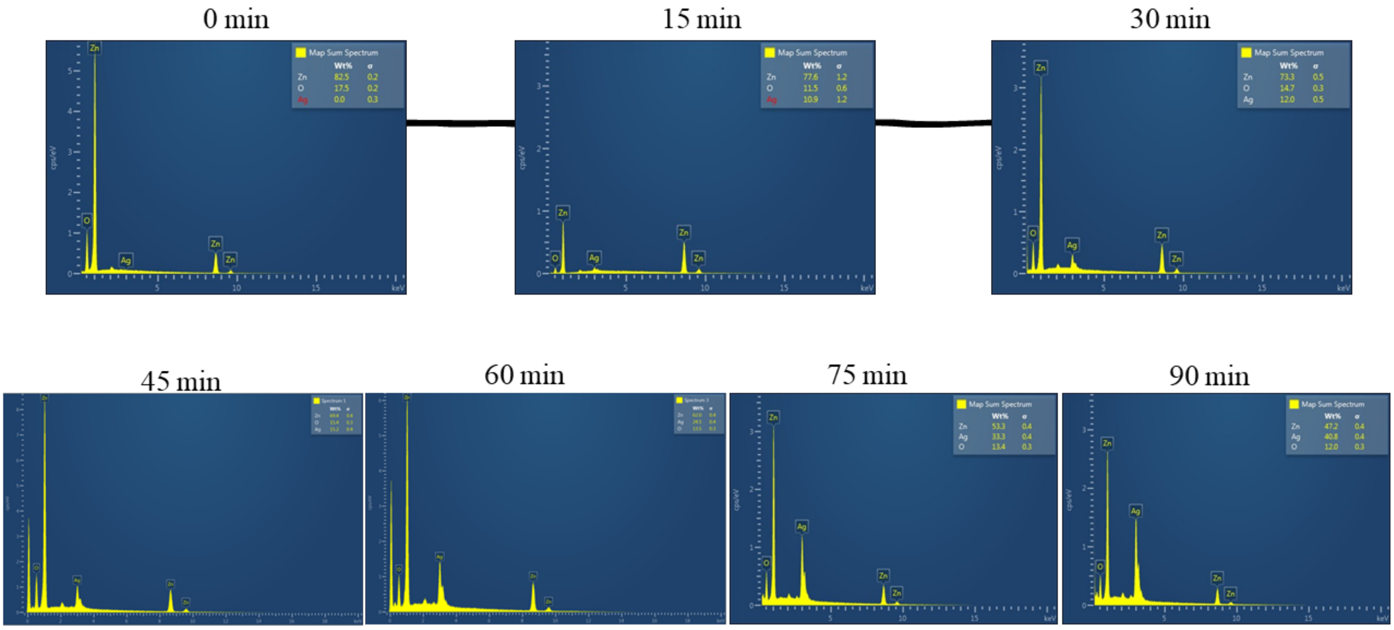

Figure S1. EDS spectrum of Ag@ZnO-ITO with various irradiation times. The EDS mapping demonstrated the proper amount of Ag NPs could be decorated on the ZnO surface via our UV synthesis process.

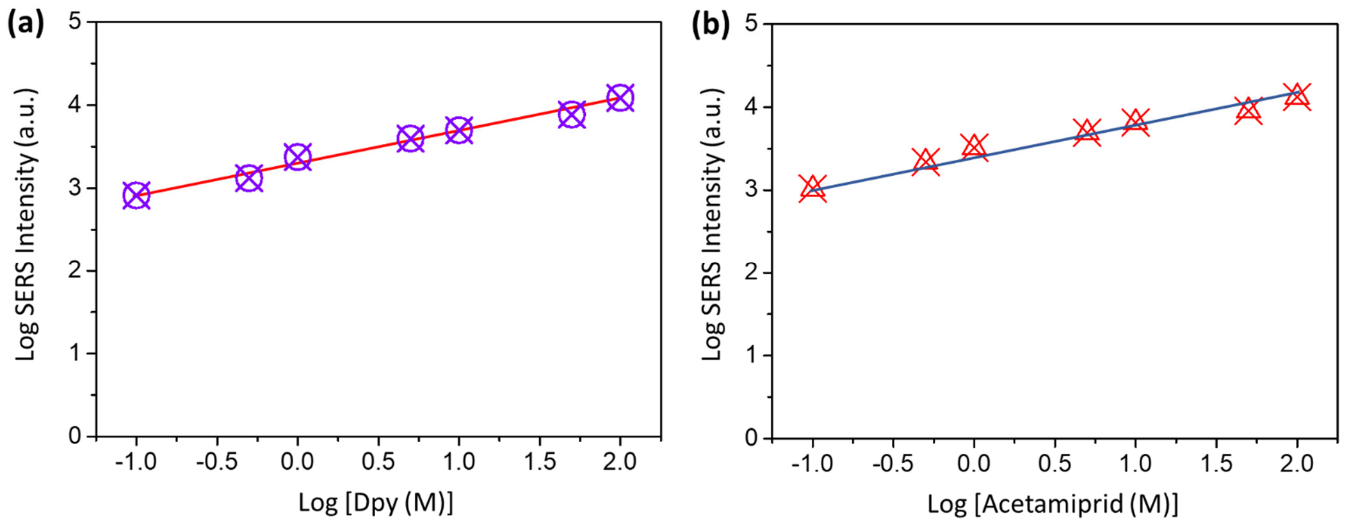

Figure S2. *EF* in the logarithmic scale of various concentrations of (a) DPY and (b) acetamiprid solution. They show a linear trend. Each graph shows a linear trend. This is the characteristic of SERS chip correlated to molecule adsorption. When the concentration is very low, the ratio of molecular adsorption is high. However, after the concentration increases, the surface becomes saturated gradually, so the proportion of molecules adsorbed decreases instead.
